# Supplementary material for: DNA-dependent protein kinase catalytic subunit (DNA-PKcs) drives chronic kidney disease progression in male mice
Source: Nat Commun. 2023 Mar 11;14:1334. doi: 10.1038/s41467-023-37043-5 (PMC10008567; doi:10.1038/s41467-023-37043-5)
Supplement: Supplementary file 1 — Supplementary Information [file 41467_2023_37043_MOESM1_ESM.pdf]

**Supplementary Information**
***for***
# DNA-dependent protein kinase catalytic subunit (DNA-PKcs) drives chronic kidney disease progression in male mice

Yunwen Yang[1,2,3], Suwen Liu[4], Peipei Wang[1,2,3], Jing Ouyang[1,2,3], Ning Zhou[1,2,3], Yue Zhang[1,2,3*]，Songming Huang[1,2,3*]，Zhanjun Jia[1,2,3*], and Aihua Zhang[1,2,3*]

[1] Department of Nephrology, Children’s Hospital of Nanjing Medical University, Guangzhou Road #72, Nanjing 210008, China

[2] Nanjing Key Laboratory of Pediatrics, Children’s Hospital of Nanjing Medical University, Nanjing 210008, China

[3] Jiangsu Key Laboratory of Pediatrics, Nanjing Medical University, Nanjing 210029, China

[4] Department of Pediatrics，Shandong Provincial Hospital Affiliated to Shandong First Medical University, Jinan, 250021, China

**Correspondence to:**

**Aihua Zhang,** Department of Nephrology, Children’s Hospital of Nanjing Medical University, 72 Guangzhou Road, Nanjing 210008, China, Tel: 0086-25-8311-7309, Fax: 0086-25-8330-4239, Email: zhaihua@njmu.edu.cn.

**Zhanjun Jia,** Department of Nephrology, Children’s Hospital of Nanjing Medical University, 72 Guangzhou Road, Nanjing 210008, China, Tel: 0086-25-8311-7309, Fax: 0086-25-8330-4239, Email: jiazj72@hotmail.com.

**Songming Huang,** Department of Nephrology, Children's Hospital of Nanjing Medical University, 72 Guangzhou Road, Nanjing 210008, China, Tel: 0086-25-8311-7309, Fax: 0086-25-8330-4239, Email: smhuang@njmu.edu.cn.

**Yue Zhang,** Department of Nephrology, Children's Hospital of Nanjing Medical University, 72 Guangzhou Road, Nanjing 210008, China, Tel: 0086-25-8311-7309, Fax: 0086-25-8330-4239, Email: zyflora2006@hotmail.com

**This supplementary file contains Supplementary Figure 1 to 8 and Supplementary Table 1 to Table 3.**

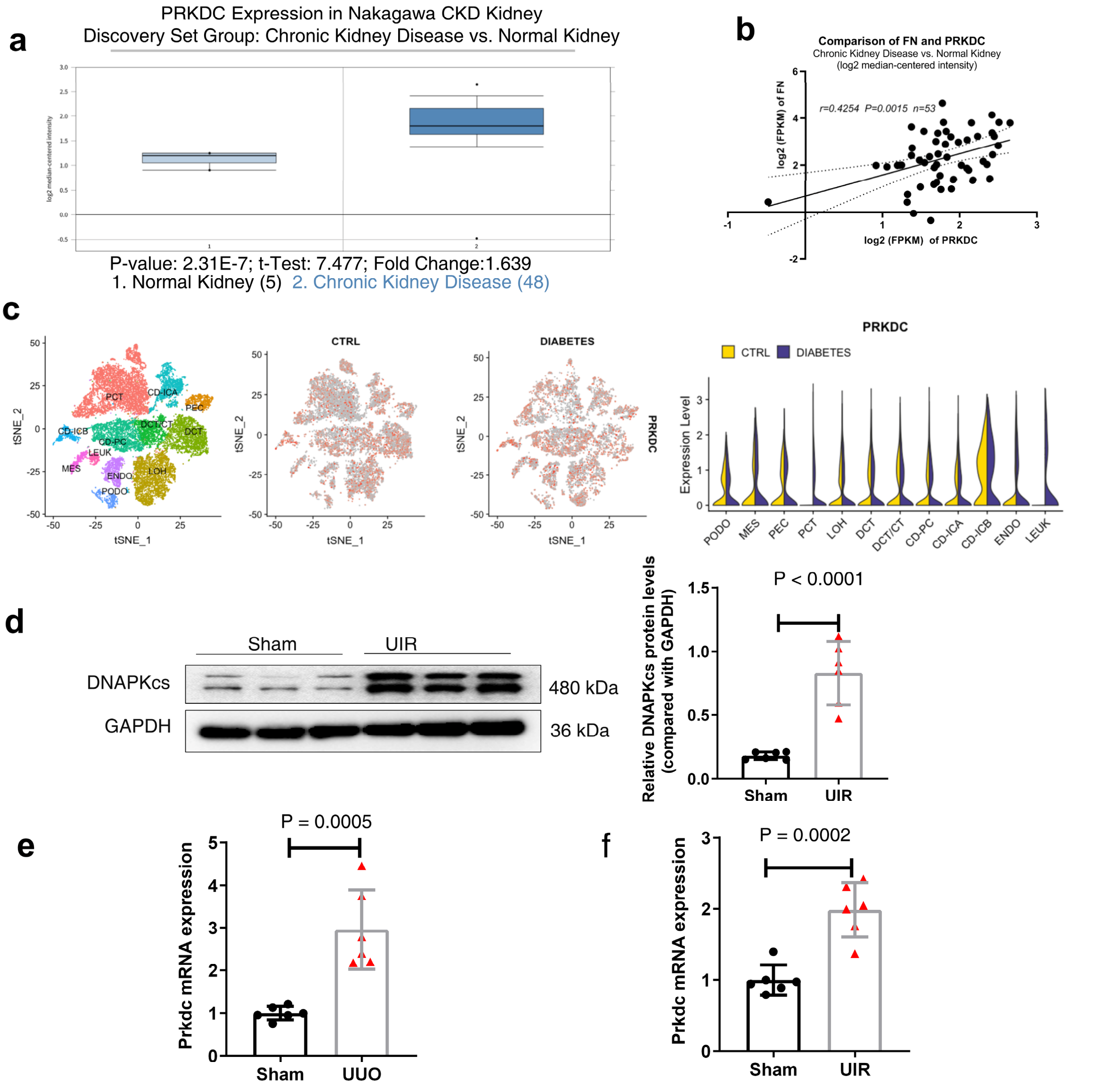


**Supplementary Fig 1. Expression of DNA-PKcs is increased in the kidneys of CKD patients and in mice with kidney fibrosis.** (a) DNA-PKcs (*Prkdc*) mRNA levels in human kidney tissues of control (n = 5) and CKD (n = 48) human study participants from Nephroseq (https://www.nephroseq.org/). Control group (Maximum: 1.242; 90th percentile: 1.242; 75th percentile: 1.242; Median: 1.19; 25th percentile: 1.054; 10th percentile: 0.911; Minimum: 0.911); CKD group (Maximum: 2.646; 90th percentile: 2.42; 75th percentile: 2.166; Median: 1.797; 25th percentile: 1.625; 10th percentile: 1.371; Minimum: -0.479). (b) Pearson's r correlation analysis between FN and DNA-PKcs mRNA levels of human study participants from Nephroseq (n=53) with 95% confidence interval from 0.1753 to 0.6239. (c) DNA-PKcs (*Prkdc*) was globally expressed in kidney cells and was upregulated in tubular epithelial cells and mesenchymal fibroblasts in human diabetic kidneys, as analyzed from a single-cell

sequencing database (http://humphreyslab.com/SingleCell/). (d) Western blot analysis of DNA-PKcs in kidney tissues of the mouse UIR model (day 21). Bars represent quantification results (mean ± SD, n=6 mice of each group). Two-tailed unpaired t-test were used to determine the p-values. QRT–PCR analysis of the mRNA levels of DNA-PKcs in kidney tissues of both the mouse UUO (day 7) model (e) and UIR (day 21) model (f). Bars represent quantification results (mean ± SD, n=6 mice of each group). Two-tailed unpaired t-test were used to determine the p-values. Source data are provided as a Source Data file.

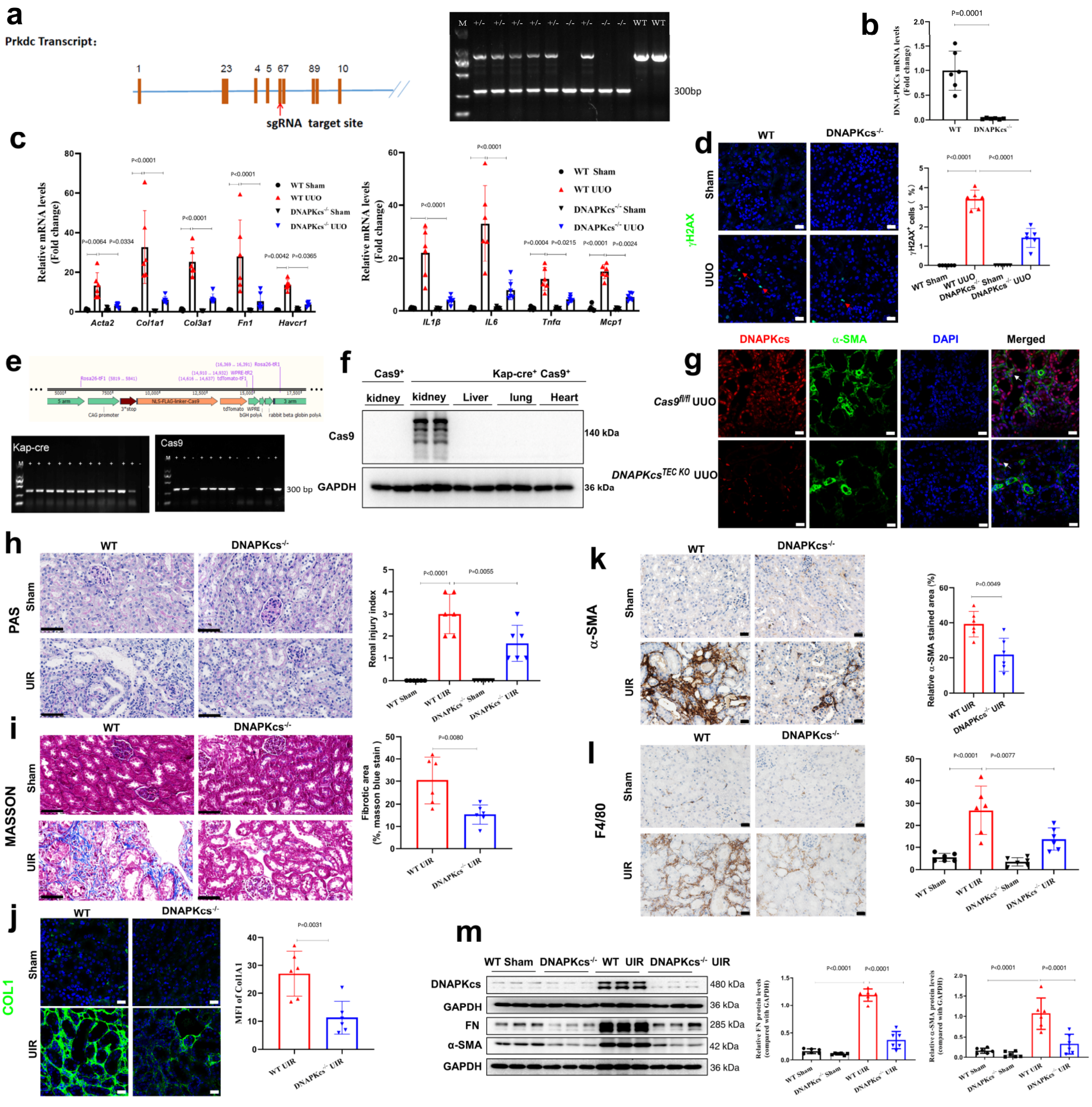


**Supplementary Fig 2. Deletion of DNA-PKcs attenuates CKD progression of in mice.** (a) Schematic depicting the generation of DNA-PKcs knockout mice and genotyping offspring by PCR analysis, wild-type (WT), heterozygous (+/-) and

homozygous (-/-). M: DNA marker. (b) qRT–PCR analysis of mRNA levels of DNA-PKcs in mouse kidneys. Bars represent quantification results (mean ± SD, n = 6 mice of each group). (c) MRNA levels of fibrosis-associated genes, *Havcr1* (KIM-1) and inflammation-associated genes were determined by qRT–PCR in kidneys of WT and DNA-PKcs$^{-/-}$ mice subjected to UUO (day 7). Bars represent quantification results (mean ± SD, n = 6 mice of each group). (d) Representative images of immunofluorescence staining for γH2AX (indicated by red arrow), scale bars: 20 μm, Bars represent quantification results (mean ± SD, n=6 mice of each group). (e) Schematic depicting the generation of CRISPR/cas9 knockin mice and genotyping Kap-Cre$^{+}$ Ca9$^{+}$ offspring by PCR analysis. M: DNA marker. (f) Protein levels of Cas9 in kidneys or other organs of Cas9$^{+}$ and Kap-Cre$^{+}$ Cas9$^{+}$ mice. N = 2 mice of each group (g) Immunofluorescence staining of DNA-PKcs and α-SMA in kidneys of DNA-PKcs $^{TEC\ KO}$ and control mice subjected to UUO (day 7), α-SMA positive myofibroblast is indicated by arrow, scale bar: 20 μm. n = 5 mice of each group. (h) Representative PAS staining of kidneys of WT and DNA-PKcs$^{-/-}$ mice subjected to UIR (day 21), scale bars: 50 μm; renal injury index analysis based on PAS staining is shown on the right. Bars represent quantification results (mean ± SD, n = 6 mice of each group). (i) Representative Masson staining of WT and DNA-PKcs$^{-/-}$ mouse kidneys subjected to UIR (scale bars: 50 μm). Bars represent quantification results (mean ± SD, n = 6 mice of each group). (j) Representative images of immunofluorescence staining for COL1, scale bars: 20 μm, blue: DAPI, green: COL1A1, Bars represent quantification results (mean ± SD, n=6 mice of each group), MFI: mean fluorescence intensity. Representative immunohistochemical staining for α-SMA (k) and F4/80 (l) in kidneys of WT and DNA-PKcs$^{-/-}$ mice subjected to UIR (scale bar: 20 μm). (m) Protein levels of DNA-PKcs, FN and α-SMA in kidneys of WT and DNA-PKcs$^{-/-}$ mice subjected to UIR were analyzed by western blot. Bars represent quantification results (mean ± SD, n = 6 mice of each group). Two-tailed unpaired t-test were used to determine the p-values for **b, i-k**. One-way ANOVA followed by Tukey's multiple comparisons test were used to determine the p-values for **d, h, l, m.** Two-way ANOVAs followed by Šídák's multiple comparisons test were used to determine the p-values for **c**. Source data are

provided as a Source Data file.

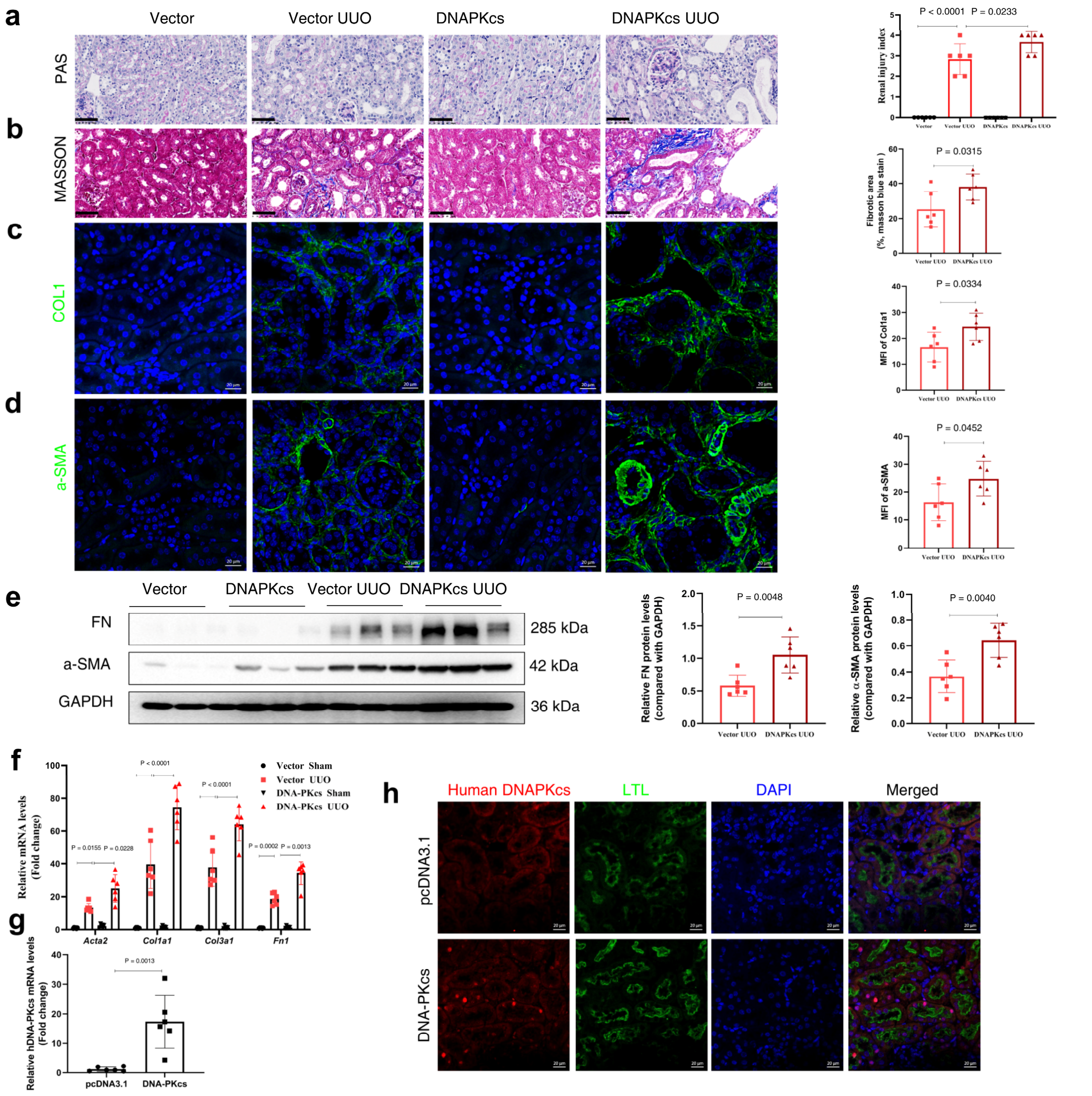


**Supplementary Fig 3**. **Ectopic expression of human DNA-PKcs is sufficient to drive the progression of renal fibrosis *in vivo*.** (a) Representative PAS staining of kidneys of mice treated with vector or human DNA-PKcs overexpression plasmids subjected to UUO (day 7), scale bars: 50 μm; Bars represent quantification results (mean ± SD, n=6 mice of each group), One-way ANOVA followed by Tukey's multiple comparisons test were used to determine the p-values. (b) Representative Masson staining of kidneys, scale bars: 50 μm; Bars represent quantification results (mean ± SD, n=6 mice of each group); Two-tailed unpaired t-test were used to determine the p-values. Representative images of immunofluorescence staining for COL1 (c) and α-SMA (d), scale bars: 20 μm, blue: DAPI, green: COL1 or α-SMA. Bars represent quantification results (mean ± SD, n=6 mice of each group), Two-tailed unpaired t-test

were used to determine the p-values, MFI: mean fluorescence intensity. (e) Protein levels of FN and α-SMA were analyzed by western blot, Bars represent quantification results (mean ± SD, n=6 mice of each group), Two-tailed unpaired t-test were used to determine the p-values. (f) MRNA levels of fibrosis-associated genes were determined by qRT–PCR in kidneys, Bars represent quantification results (mean ± SD, n=6 mice of each group), Two-way ANOVAs followed by Šídák's multiple comparisons test were used to determine the p-values. (g) MRNA levels of human DNA-PKcs were determined by qRT–PCR in kidneys with injection of vector or human DNA-PKcs overexpression plasmids for 36 h, Bars represent quantification results (mean ± SD, n=6 mice of each group), Two-tailed unpaired t-test were used to determine the p-values. (h) Immunofluorescence staining of ectopic human DNA-PKcs by using a human special antibody in kidneys with injection of vector or human DNA-PKcs overexpression plasmids for 36 h, scale bars: 20 μm, n = 3 mice of each group. Source data are provided as a Source Data file.

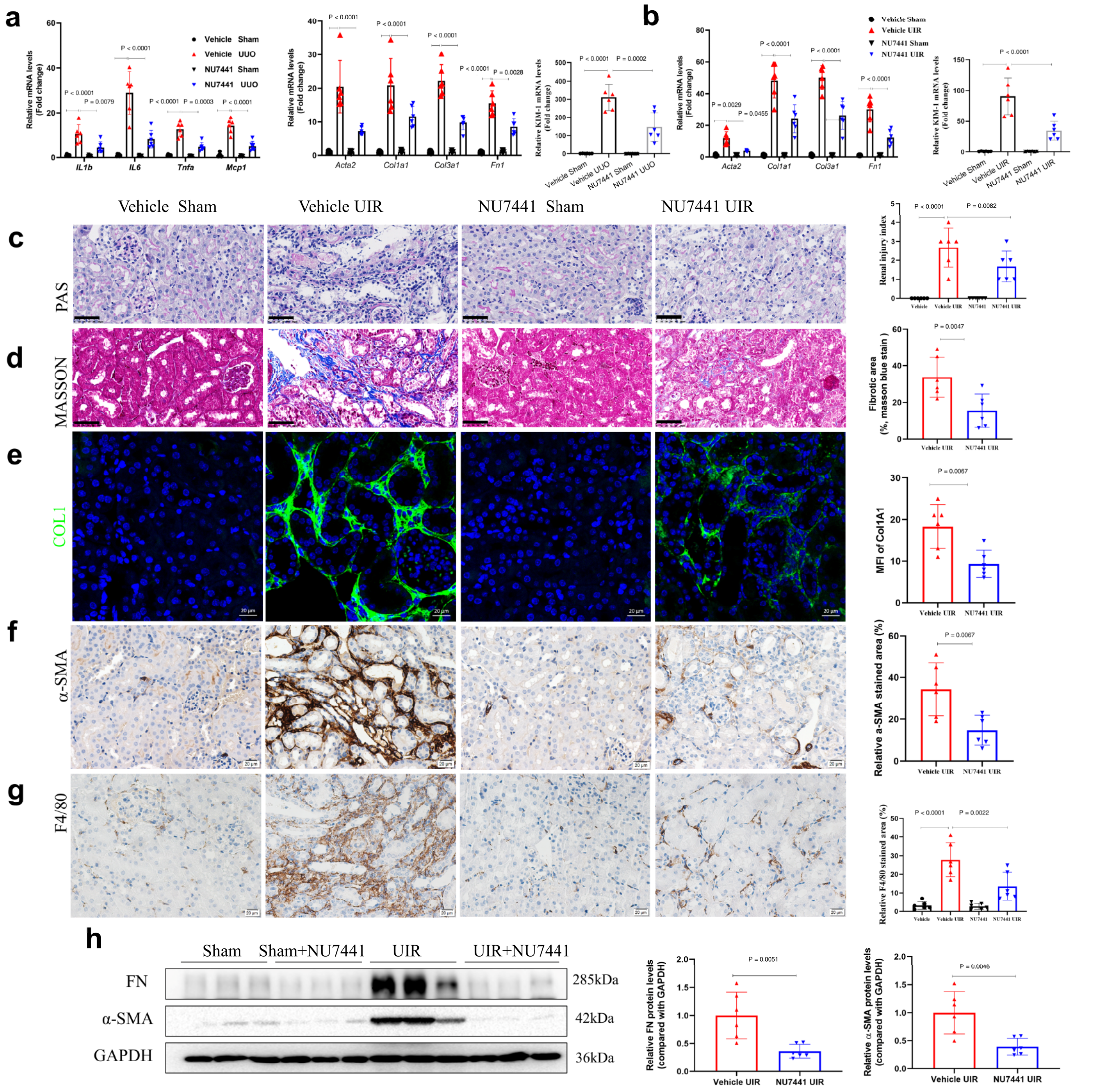

**Supplementary Fig 4. Inhibition of DNA-PK activity attenuates the development of CKD in UUO or UIR mice.** (a) MRNA levels of fibrosis-associated genes, *Havcr1* (KIM-1) and inflammation-associated genes determined by qRT–PCR in kidneys of mice treated with NU7441 (40 mg/kg) or vehicle subjected to UUO (day 7). Bars represent quantification results (mean ± SD, n=6 mice of each group). (b) MRNA levels of fibrosis-associated genes and *Havcr1* (KIM-1) were determined by qRT–PCR in kidneys of mice treated with nu7441 or vehicle subjected to UIR (day 21). Bars represent quantification results (mean ± SD, n=6 mice of each group). (c) Representative PAS staining of kidneys from mice treated with nu7441 or vehicle subjected to UIR (day 21), scale bars: 50 μm; Bars represent quantification results (mean ± SD, n=6 mice of each group). (d) Representative Masson staining of kidneys, scale bars: 50 μm. Bars represent quantification results (mean ± SD, n=6 mice of each

group). (e) Representative images of immunofluorescence staining for COL1 in kidneys from NU7441 (40 mg/kg) or vehicle-treated mice, scale bars: 20 μm, blue: DAPI, green: COL1. Bars represent quantification results (mean ± SD, n=6 mice of each group). Representative immunohistochemical staining of α-SMA (f) and F4/80 (g) in the kidneys of NU7441 (40 mg/kg) or vehicle-treated mice (scale bars: 20 μm). Bars represent quantification results (mean ± SD, n=6 mice of each group). (h) Protein levels of FN and α-SMA in the kidneys of NU7441- or vehicle-treated mice subjected to UIR were analyzed by western blot. Bars represent quantification results (mean ± SD, n=6 mice of each group). One-way ANOVA followed by Tukey's multiple comparisons test were used to determine the p-values for **c-h &** KIM-1 in **a, b**, Two-way ANOVAs followed by Šídák's multiple comparisons test were used to determine the p-values for **a, b** except KIM-1. Source data are provided as a Source Data file.

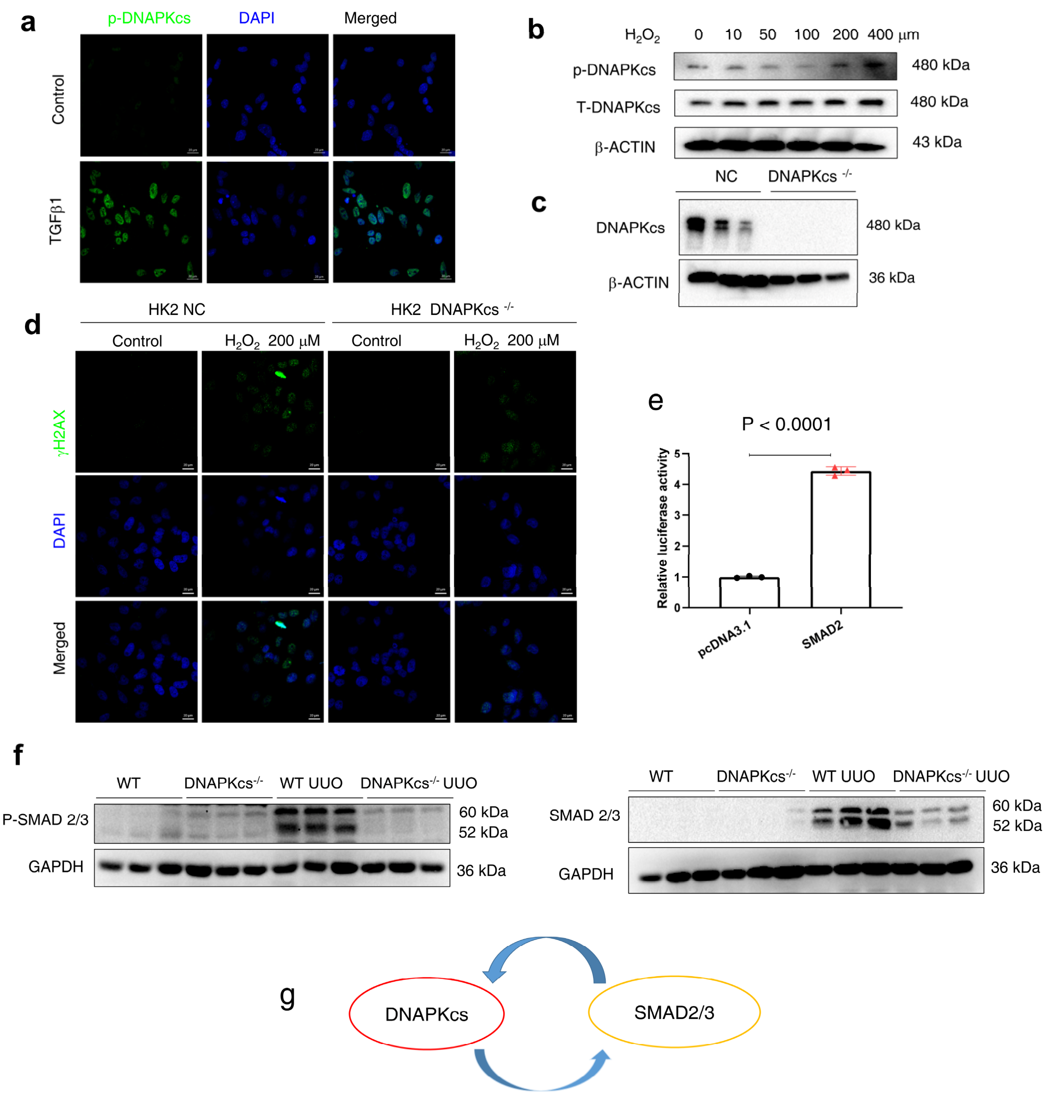


**Supplementary Fig 5. Expression of DNA-PKcs is induced by $H_2O_2$ or TGFβ1-SMAD.** (a) Representative immunofluorescence images of p-DNA-PKcs in primary tubular epithelial cells treated with TGFβ1 (5 ng/ml) for 24 h, scale bars: 20 μm. n=3 biologically independent experiments. (b) Western blot analysis of protein levels of p-DNA-PKcs (S2056) and total DNA-PKcs in HK-2 cells treated with $H_2O_2$ for 1 h at different concentrations as indicated. n=3 biologically independent experiments. (c) Successful construction of DNA-PKcs knockout HK-2 cells confirmed by western blot analysis. n=3 biologically independent experiments. (d) Representative images of immunofluorescence staining for γH2AX, scale bars: 20 μm. n=3 biologically independent experiments. (e) Luciferase reporter assay of DNA-PKcs promoter in HEK293T cells with SMAD2 overexpression for 24 h. Bars represent quantification results (mean ± SD, n = 3 biologically independent experiments). Two-tailed unpaired

t-test were used to determine the p-values. (f) Protein levels of SMAD2, SMAD3 and phosphorylated-SMAD2/SMAD3 were analyzed by western blot in kidney tissues of DNA-PKcs$^{-/-}$ and WT control mice subjected to UUO (day 7). n = 6 mice of each group. (g) A loop working model of SMAD-DNA-PKcs signaling. NC: negative control. Source data are provided as a Source Data file.

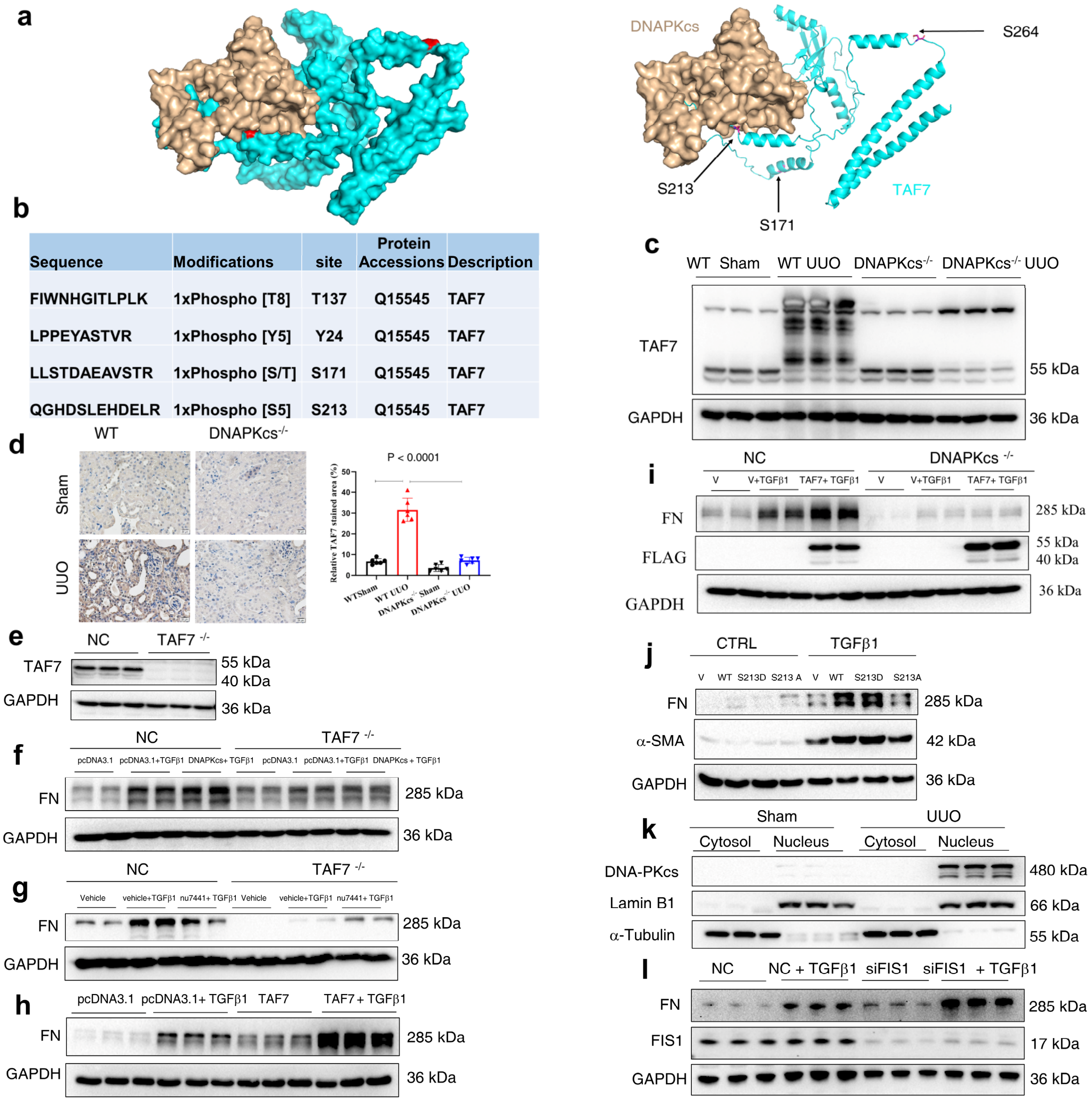


**Supplementary Fig 6. DNA-PK-mediated phosphorylation of TAF7 aggravates TGFβ1-induced tubular epithelial cell dedifferentiation and fibroblast activation.** (a) Protein-Protein docking between DNA-PKcs and TAF7 was performed through an online database ClusPro to analysis their possible direct interaction. The docking result

showed a possible direct interaction between DNA-PKcs and TAF7, 213 site serine of TAF7 is one of the nearest sites perhaps binds to DNA-PKcs. (b) The results of mass spectrometry showed S213, T137, Y24 and S171 sites of human TAF7 were phosphorylated by DNA-PKcs. (c) The phosphorylation of TAF7 in UUO mice was also confirmed by Phos-tag SDS-PAGE. n = 3 mice of each group (d) Representative immunohistochemical staining of TAF7 in the kidneys of UUO (day 7) mice, scale bars: 20 μm. Bars represent quantification results (mean ± SD, n = 6 mice of each group). Two-tailed unpaired t-test were used to determine the p-values, One-way ANOVA followed by Tukey's multiple comparisons test were used to determine the p-values. (e) Successful construction of TAF7 knockout mPTCs was confirmed by western blot analysis, n=3 biologically independent experiments. Western blot analysis of protein levels of FN in $TAF7^{-/-}$ mPTCs transfected with DNA-PKcs overexpression plasmids (f) or treated with 0.1 μM NU7441 (g) and then treated with TGFβ1 for 24 h. n=3 biologically independent experiments. (h) western blot analysis of protein levels of FN in HK-2 cells transfected with TAF7 overexpression plasmids or vector and then treated with TGFβ1 for 24 h, n=3 biologically independent experiments. (i) After transfection with FLAG-tagged TAF7 in both $DNA\text{-}PKcs^{-/-}$ and NC HK-2 cells, protein levels of FN and TAF7 were analyzed by western blot, n=3 biologically independent experiments. (j) After transfection with TAF7 mutants TAF7-WT, TAF7-S213D and TAF7-S213A in NRK-49F cells, the protein levels of FN and α-SMA were analyzed by western blot, n=3 biologically independent experiments. (k) The expression and subcellular location of DNA-PKcs were measured in kidneys after UUO for 7 days through fractionated

western blotting, n = 6 mice of each group. (l) Protein levels of FN and FIS1 were analyzed by western blot in HK2 cells transfected with si-NC or si-FIS1 then treated with TGFβ1 (5 ng/ml) for 24 h, n=3 biologically independent experiments. NC: negative control. CTRL: control. Source data are provided as a Source Data file.

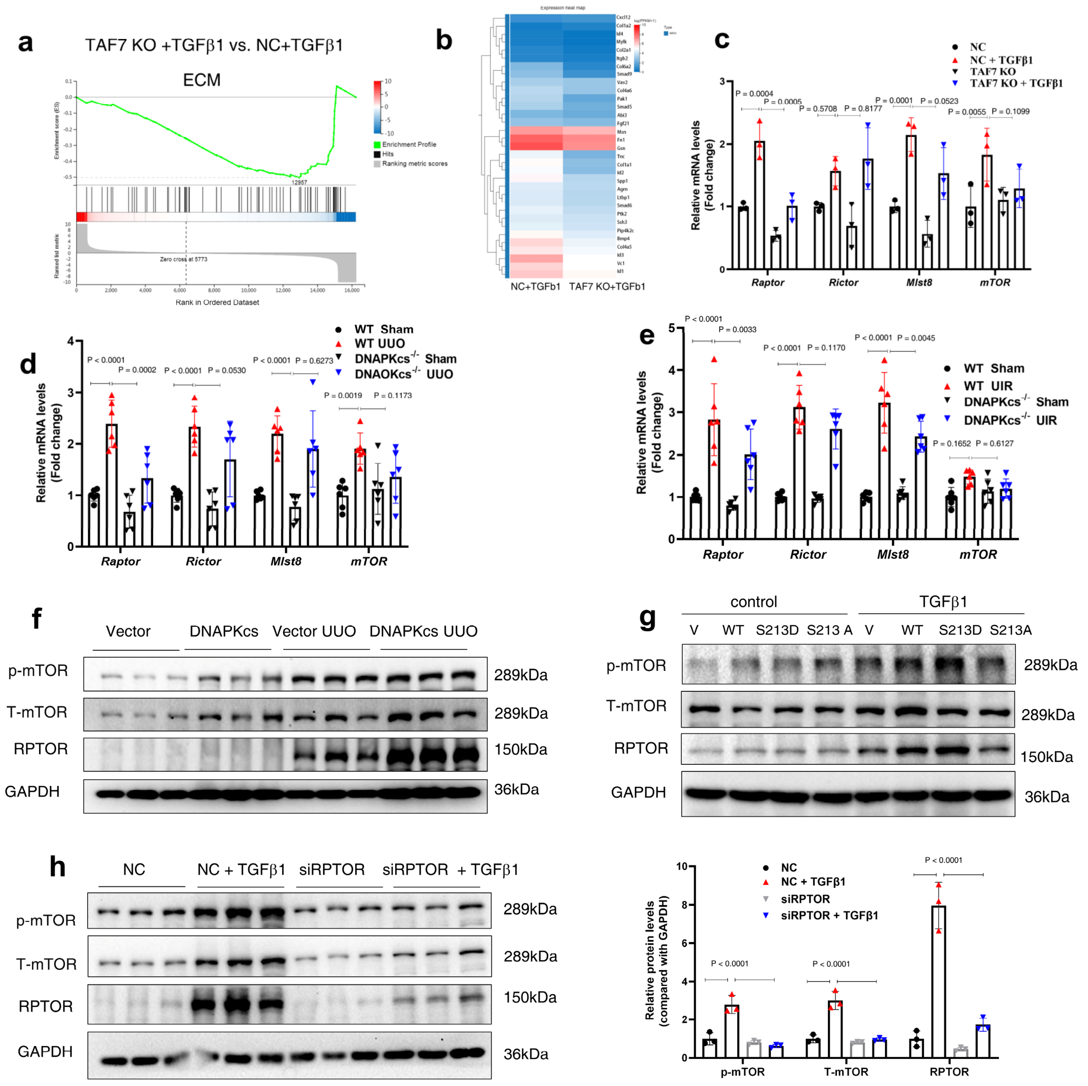


**Supplementary Fig 7. DNA-PKcs mediates mTORC1 activation by upregulating Raptor expression.** (a) GSEA enrichment (n = 2 biologically independent samples of each group) and heatmap analysis (b) showing that TAF7 knockout ameliorated TGFβ1-induced fibrosis-associated gene expression in mPTCs; ECM (extracellular matrix). MRNA levels of mTOR and its protein partners, including *Raptor, Rictor* and

*Mlst8*, were analyzed by qRT–PCR in TAF7$^{-/-}$ and NC mPTCs treated with TGFβ1 (c), Bars represent quantification results (mean ± SD, n = 3 biologically independent experiments) and in kidney tissues of DNA-PKcs$^{-/-}$ and WT control mice subjected to UUO (d) or UIR (e), Bars represent quantification results (mean ± SD, n = 6 mice of each group). (f) Protein levels of RPTOR, mTOR and phosphorylated mTOR were analyzed by western blot in kidney tissues of DNA-PKcs overexpression or control group mice, n = 6 mice of each group. (g) Protein levels of RPTOR, mTOR and phosphorylated mTOR were analyzed by western blot in HK-2 cells transfected with TAF7 mutants, n = 3 biologically independent experiments. (h) Protein levels of RPTOR, mTOR and phosphorylated mTOR were analyzed by western blot in HK-2 cells transfected with si-NC or si-RPTOR then treated with TGFβ1(5 ng/ml) for 24 h, KD: knockdown. Bars represent quantification results (mean ± SD, n = 3 biologically independent experiments), Two-way ANOVAs followed by Šídák's multiple comparisons test were used to determine the p-values for **c-h**. NC: negative control. Source data are provided as a Source Data file.

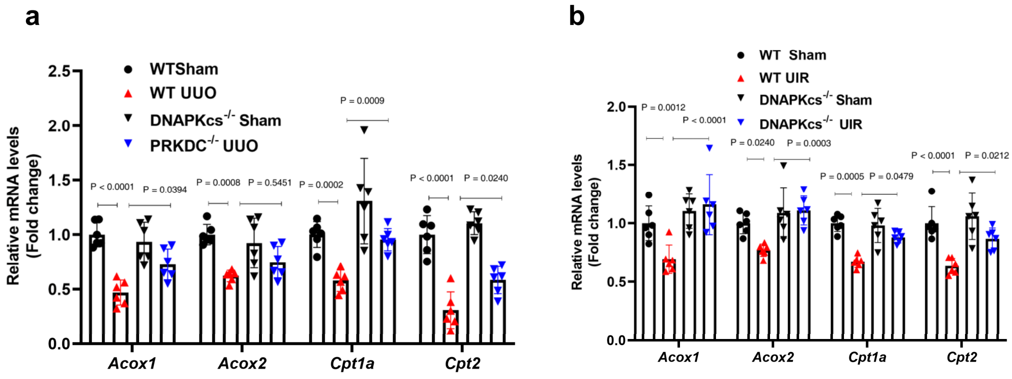


**Supplementary Fig 8. DNA-PKcs knockout corrects metabolic reprogramming.** MRNA levels of FAO pathway-associated enzymes analyzed by qRT-PCR in kidney tissues of DNA-PKcs$^{-/-}$ and WT control mice subjected to UUO (day 7) (a) or UIR (day 21) (b). Bars represent quantification results (mean ± SD, n = 6 mice of each group). Two-way ANOVAs followed by Šídák's multiple comparisons test were used to determine the p-values. Source data are provided as a Source Data file.

**Supplementary Table 1. Clinical data of CKD patients.**

| Diagnosis | Age (1-10 years old) | Age (11-15 years old) | Total |
|---|---|---|---|
| IgA nephropathy | 1 female | 1 female | 2 |
| Crescent glomerulonephritis | 1 female | 0 | 1 |
| Glomerulonephritis | 2 males | 2 males | 4 |
| LN IV-G(A/C) +V | 0 | 1 female | 1 |
| FSGS | 1 female | 1 female | 2 |
| HSPN III-a | 0 | 1 male | 1 |
| Subacute tubulointerstitial nephropathy | 1 male | 0 | 1 |
| LN | 1 female | 1 female | 2 |
| Refractory nephrotic syndrome | 1 female | 0 | 1 |

15 male and female CKD patients with tubulointerstitial fibrosis. **Abbreviation**: LN: Lupus nephritis; HSPN: Henoch-Schonlein purpura nephritis; FSGS: Focal segmental glomerular sclerosis

**Supplementary Table 2. Sequences of oligonucleotides**

| Gene | Primer Sequence (5'-3') |
|---|---|
| Mouse *Il-6* | F: ACAAAGCCAGAGTCCTTCAGAGAG |
| | R: TTGGATGGTCTTGGTCCTTAGCCA |
| Mouse *Mcp-1* | F: GCTCTCTCTTCCTCCACCAC |
| | R: ACAGCTTCTTTGGGACACCT |
| Mouse *Il-1β* | F: ACTGTGAAATGCCACCTTTTG |
| | R: TGTTGATGTGCTGCTGTGAG |
| Mouse *Tnf-α* | F: TCCCCAAAGGGATGAGAAG |
| | R: CACTTGGTGGTTTGCTACGA |
| Mouse *Prkdc* | F: AAACCTGTTCCGAGCTTTTCTG |
| | R: TCTCAATCTGAGGACGAATTGC |
| Mouse *Gapdh* | F: GTCTTCACTACCATGGAGAAGG |
| | R: TCATGGATGACCTTGGCCAG |
| Mouse *Col1a1* | F: CCGGCTCCTGCTCCTCTT |
| | R: TTGCACGTCATCGCACAC |
| Mouse *Col3a1* | F: TGGTTTCTTCTCACCCTTCTT |
| | R: CAAATGGGATCTCTGGGTTG |
| Mouse *Fn1* | F: CGTGGAGCAAGAAGGACAA |
| | R: GTGAGTCTGCGGTTGGTAAA |
| Mouse *Acta2* | F: CCCTGAAGAGCATCCGACA |
| | R: CCAGAGTCCAGCACAATACC |
| Mouse *Havcr1* | F: ACATATCGTGGAATCACAACGAC |
| | R: ACTGCTCTTCTGATAGGTGACA |
| Mouse *Rptor* | F: CAGTCGCCTCTTATGGGACTC |
| | R: GGAGCCTTCGATTTTCTCACA |
| Mouse *Rictor* | F: GCTGCGCTATCTCATCCAAGA |
| | R: GGGTTCTGAAGTGCTAGTTCAC |
| Mouse *Mlst8* | F: CCCCATCATCAGTTATGACGG |
| | R: CCTGGAAGATACGCTGACACT |

| | |
|---|---|
| Mouse *mTOR* | F: ACCGGCACACATTTGAAGAAG |
| | R: CTCGTTGAGGATCAGCAAGG |
| Mouse *Cpt1a* | F: GGTCTTCTCGGGTCGAAAGC |
| | R: TCCTCCCACCAGTCACTCAC |
| Mouse *Cpt2* | F: CAGCACAGCATCGTACCCA |
| | R: TCCCAATGCCGTTCTCAAAAT |
| Mouse *Acox1* | F: TAACTTCCTCACTCGAAGCCA |
| | R: AGTTCCATGACCCATCTCTGTC |
| Mouse *Acox2* | F: CACCCTGACATAGACAGTGAAAG |
| | R: CTGGGTCACGTTGGATGAGG |
| Human *Prkdc* | F: AGCTGGCTTGCGCCTATTT |
| | R: GGGCACACCACTTTAACAAGAC |
| sgRNA (SpCas9) | |
| Human DNA-PKcs sgRNA-1 | F: CACCGAAAACGACAAACGCGGTCGG |
| | R: AAACCCGACCGCGTTTGTCGTTTTC |
| Human DNA-PKcs sgRNA-2 | F: CACCGCCGGTCATCAACTGATCCG |
| | R: AAACCGGATCAGTTGATGACCGGC |
| Mouse DNA-PKcs sgRNA | F: CACCGAACTTCACCTAATACTCCC |
| | R: AAACGGGAGTATTAGGTGAAGTTC |
| Human KU80 sgRNA | F: CACCGCATGGCCACCTAAGCGAAAG |
| | R: AAACCTTTCGCTTAGGTGGCCATGC |
| Mouse TAF7 sgRNA-1 | F: CACCGTATAGGTCACCATCTACTG |
| | R: AAACCAGTAGATGGTGACCTATAC |
| Mouse TAF7 sgRNA-2 | F: CACCGCTCCTACAACAATTAAAGG |
| | R: AAACCCTTTAATTGTTGTAGGAGC |
| Rat TAF7 sgRNA-1 | F: CACCGTATAGATCACCATCTACTG |
| | R: AAACCAGTAGATGGTGATCTATAC |
| Rat TAF7 sgRNA-2 | F: CACCGTCTACCAATTTTGCAGCCAG |
| | R: AAACCTGGCTGCAAAATTGGTAGAC |

| | |
|---|---|
| Luciferase reporter Promoter | |
| Human *Rptor* | F: TCCCAGGTCACAGAGCTAG |
| | R: TGGCCGACAGACCAAACCTC |
| Human *Prkdc* | F: TACCCGAGAGCTGGGAGTGC |
| | R: AGGAACTTTCCCGGGGACC |
| ChIP assay primers | |
| Human *Rptor* | F: GTAACCGAGGTAACGGGGTC |
| | R: GACCAAACCTCCTCAGAGCG |
| si-human *RPTOR* | 5'-GGACAACGGCCACAAGUAC-3' |
| si-human *Fis1* | 5'-GGCUCAAGGAAUACGAGAAGG-3 |
| Genotyping | |
| DNA-PKcs KO | Prkdc-GT-tF5: GGCTGGTCCTCAAACCATG |
| | Prkdc-GT-tR5: CACCATCCCTCTTAAGTGC |
| Kap cre | F: AGATGCCAGGACATCAGGAACCTG |
| | R: ATCAGCCACACCAGACACAGAGATC |
| Cas9 | tdTomato-tF1: CGGCATGGACGAGCTGTACAAG |
| | WPRE-tR2: TCAGCAAACACAGTGCACACCAC |

**Supplementary Table 3. ANTIBODIES AND REAGENTS TABLE**

| Antibodies | SOURCE | clone | Dilution | IDENTIFIER |
|---|---|---|---|---|
| Rabbit monoclonal anti-DNA-PKcs | Abcam | Y393 | 1:1000 | Cat#ab32566 |
| Rabbit polyclonal anti-DNA-PKcs (phospho S2056) | Abcam | | 1:1000 | Cat#ab18192 |
| Rabbit monoclonal anti-DNA-PKcs (human) | Abcam | EPR392 | 1:1000 | Cat#ab133516 |
| Rabbit polyclonal anti-DNA-PKcs | Abcam | | 1:100 | Cat#ab70250 |
| Rabbit polyclonal anti-Fibronectin | Abcam | | 1:1000 | Cat#ab2413 |
| Rabbit monoclonal anti-FLAG (ChIP) | CST | D6W5B | 1:1000 | Cat#14793 |
| Rabbit monoclonal anti-Cas9 | CST | E7M1H | 1:1000 | Cat#19526 |
| Rabbit monoclonal anti-α-Smooth Muscle Actin | CST | D4K9N | 1:1000 | Cat#19245 |
| Rabbit monoclonal anti-COL1A1 | CST | E8F4L | 1:1000 | Cat#72026 |
| Rabbit monoclonal anti-Raptor | CST | 24C12 | 1:1000 | Cat#2280 |
| Rabbit monoclonal anti-mTOR (phospho S2448) | CST | D9C2 | 1:1000 | Cat#5536 |
| Rabbit polyclonal anti-mTOR | CST | | 1:1000 | Cat#2972 |
| Rabbit monoclonal anti-F4/80 | CST | D2S9R | 1:150 | Cat#70076 |
| Rabbit monoclonal anti-Histone H2A.X (Ser139) | CST | 20E3 | 1:200 | Cat#9718 |

| | | | | |
|---|---|---|---|---|
| Mouse monoclonal anti-α-Smooth Muscle Actin | CST | 1A4 | 1:200 | Cat#48938 |
| Mouse monoclonal anti-TAF7 | Santa Cruz | SQ-8 | 1:1000 | Cat#sc101167 |
| Mouse monoclonal anti-Flag-tag | Sigma-Aldrich | M2 | 1:1000 | Cat#F1804 |
| Mouse monoclonal anti-GAPDH | Proteintech | 1E6D9 | 1:1000 | Cat#60004-1-Ig |
| Mouse monoclonal anti-β-ACTIN | Proteintech | 2D4H5 | 1:1000 | Cat#66009-1-Ig |
| Rabbit polyclonal anti-ACOX1 | Proteintech | | 1:1000 | Cat#10957-1-AP |
| Rabbit polyclonal anti-CPT1A | Proteintech | | 1:1000 | Cat#15184-1-AP |
| Rabbit polyclonal anti-LDHA | Proteintech | | 1:1000 | Cat#19987-1-AP |
| Rabbit polyclonal anti-FIS1 | Proteintech | | 1:1000 | Cat#10956-1-AP |
| Rabbit polyclonal anti-Lamin B1 | Proteintech | | 1:1000 | Cat#12987-1-AP |
| Mouse monoclonal anti-Alpha Tubulin | Proteintech | 1E4C11 | 1:1000 | Cat#66031-1-Ig |
| Mouse monoclonal anti-HK2 | Proteintech | 2A11C3 | 1:1000 | Cat#66974-1-Ig |
| Goat anti-rabbit IgG-HRP | Beyotime | | 1:1000 | Cat#A0208 |
| Goat anti-mouse IgG-HRP | Beyotime | | 1:1000 | Cat#A0216 |
| Donkey anti-Rabbit IgG (H+L) Highly Cross-Adsorbed Secondary Antibody, Alexa Fluor 488 | Thermo Fisher Scientific | | 1:500 | Cat#21206 |
| Donkey anti-Rabbit IgG | Thermo | | 1:500 | Cat#A32794 |

| | | | |
|---|---|---|---|
| (H+L) Highly Cross-Adsorbed Secondary Antibody, Alexa Fluor™ Plus 555 | Fisher Scientific | | |
| LTL | Vector lab | 1:200 | Cat#FL1321 |
| REAGENT or RESOURCE | SOURCE | | IDENTIFIER |
| Boost IHC Detection Reagent (HRP, Rabbit) | Cell Signaling Technology | | Cat#8114S |
| Protein A/G PLUS-Agarose | Santa Cruz Biotechnology | | Cat#sc-2003 |
| Normal mouse IgG | Santa Cruz Biotechnology | | Cat#sc-2025 |
| Chemicals and recombinant proteins | SOURCE | | IDENTIFIER |
| NU7441 | MedChemExpress | | Cat#HY-11006 |
| PEG400 | MedChemExpress | | Cat#HY-Y0873A |
| Phosbind Acrylamide | APExBIO | | Cat#F4002 |
| Penicillin/Streptomycin | GIBCO | | Cat#15140-122 |
| Collagenase type II | GIBCO | | Cat#17101015 |
| BD Matrigel | BD Biocoat | | Cat# 356234 |
| Fetal Bovine Serum (FBS) | GIBCO | | Cat#10099141C |
| Penicillin/Streptomycin | GIBCO | | Cat#15140-122 |
| Recombinant Human TGF-beta 1 | R&D Systems | | Cat#240-B-002 |
| Recombinant Murine EGF | Peprotech | | Cat# 315-09 |
| DAPI | Beyotime | | Cat#C1005 |
| Hoechst 33342 | Beyotime | | Cat#C1028 |
| $H_2O_2$ | Beyotime | | Cat#S0038 |
| GST-TAF7 Fusion Protein | Proteintech | | Cat#Ag4406 |
| Protease inhibitor cocktail | Roche | | Cat#04693132001 |

| | | |
|---|---|---|
| Phosphatase inhibitor | Roche | Cat#4906837001 |
| Critical commercial assays | SOURCE | IDENTIFIER |
| PrimeScript 1st Strand cDNA Synthesis Kit | TAKARA | Cat# H6110A |
| ChamQ SYBR Color qPCR Master Mix (Low ROX Premixed) | Vazyme | Cat#Q431-02 |
| BCA Protein assay kit | Beyotime | Cat#P0012 |
| 3,3'-Diaminobenzidine (DAB) substrate kit | Proteintech | Cat#PK10005 |
| SimpleChIP® Enzymatic Chromatin IP Kit (Magnetic Beads) | Cell Signaling Technology | Cat#9003 |
| ClonExpress® Ultra One Step Cloning Kit | Vazyme | Cat#C115-01 |
| Dual-Luciferase® Reporter Assay System | Promega | Cat#E1910 |
| TRIzol reagent | Life Technologies | Cat#15596018 |
| L-Lactic Acid/Lactate (LA) Colorimetric Assay Kit | Elabscience | Cat#E-BC-K044-S |
| Experimental models: Cell lines | SOURCE | IDENTIFIER |
| Human: HK-2 | ATCC | Cat#CRL-2190 |
| Mouse: mPTC | ATCC | Cat#CRL-3361 |
| Rat: NRK-49F | ATCC | Cat#CRL-1570 |
| Human 293T | ATCC | Cat# CRL-11268 |
| Experimental models: Organisms/strains | SOURCE | IDENTIFIER |
| Mouse: global DNA-PKcs | This paper, Gempharmatech | Cat#T001455 |

| | | |
|---|---|---|
| KO mice | | |
| Mouse: Rosa26-LSL-Cas9-tdTomato | This paper, Gempharmatech | Cat#T002249 |
| Mouse: Kap-icre | Jackson | Cat#008781 |
| Recombinant DNA | SOURCE | IDENTIFIER |
| pcDNA-Flag-human TAF7 | GeneCopoeia | Cat#F1001 |
| pcDNA-Flag-human TAF7 $S^{213}D$ | This paper | N/A |
| pcDNA-Flag-human TAF7 $S^{213}A$ | This paper | N/A |
| Human DNA-PKcs | Addgene | Cat# 83317 |
| pGL3 basic | Promega | Cat#U47295 |
| PRL | Promega | Cat#AF025846 |
| Continued | | |
| Human Raptor promoter | This paper | N/A |
| Human DNA-PKcs promoter | This paper | N/A |
| Human SMAD2 | GeneCopoeia | Cat#M0679 |
| pSpCas9(BB)-2A-Puro (PX459) V2.0 | Addgene | Cat# 62988 |
| PX459-human DNA-PKcs sgRNA | This paper | N/A |
| PX459-mouse TAF7 sgRNA | This paper | N/A |
| PX459-Rat TAF7 sgRNA | This paper | N/A |
| Software and Algorithms | SOURCE | IDENTIFIER |
| GraphPad Prism 9.0 | Graphpad | https://www.graphpad.com/ |
| Image Lab Software | Bio-Rad | https://www.bio-rad.com/en- |

| | | |
|---|---|---|
| | | cn/product/image-lab-software |
| ZEN 2010 software | Carl Zeiss | https://www.zeiss.com/microscopy/us/products/microscope-software/zen-lite.html |
| Benchling | Benchling Sync | https://benchling.com/ |
| ImageJ | NIH | https://imagej.nih.gov/ij/ |

| other | SOURCE | IDENTIFIER |
|---|---|---|
| PVDF membrane | Millipore | Cat#IPVH00010 |
| non-traumatic microaneurysm clamp | RWD Life Science | Cat#R31005-06 |
