# Supplementary material for: DNA-dependent protein kinase catalytic subunit (DNA-PKcs) drives chronic kidney disease progression in male mice
Source: Nat Commun. 2023 Mar 11;14:1334. doi: 10.1038/s41467-023-37043-5 (PMC10008567; doi:10.1038/s41467-023-37043-5)
Supplement: Supplementary file 3 — Reporting Summary [file 41467_2023_37043_MOESM3_ESM.pdf]

nature portfolio

Corresponding author(s): Zhanjun Jia

Last updated by author(s): Feb 18, 2023

Reporting Summary

Nature Portfolio wishes to improve the reproducibility of the work that we publish. This form provides structure for consistency and transparency in reporting. For further information on Nature Portfolio policies, see our Editorial Policies and the Editorial Policy Checklist.

Statistics

For all statistical analyses, confirm that the following items are present in the figure legend, table legend, main text, or Methods section.

n/a	Confirmed	
☐	☒	The exact sample size (*n*) for each experimental group/condition, given as a discrete number and unit of measurement
☐	☒	A statement on whether measurements were taken from distinct samples or whether the same sample was measured repeatedly
☐	☒	The statistical test(s) used AND whether they are one- or two-sided<br>*Only common tests should be described solely by name; describe more complex techniques in the Methods section.*
☒	☐	A description of all covariates tested
☐	☒	A description of any assumptions or corrections, such as tests of normality and adjustment for multiple comparisons
☐	☒	A full description of the statistical parameters including central tendency (e.g. means) or other basic estimates (e.g. regression coefficient) AND variation (e.g. standard deviation) or associated estimates of uncertainty (e.g. confidence intervals)
☐	☒	For null hypothesis testing, the test statistic (e.g. *F*, *t*, *r*) with confidence intervals, effect sizes, degrees of freedom and *P* value noted<br>*Give P values as exact values whenever suitable.*
☒	☐	For Bayesian analysis, information on the choice of priors and Markov chain Monte Carlo settings
☒	☐	For hierarchical and complex designs, identification of the appropriate level for tests and full reporting of outcomes
☐	☒	Estimates of effect sizes (e.g. Cohen's *d*, Pearson's *r*), indicating how they were calculated

Our web collection on statistics for biologists contains articles on many of the points above.

Software and code

Policy information about availability of computer code

Data collection	No custom computer code or algorithm was used in this study.
Data analysis	The data were analyzed using Graphpad Prism (v9.0), mage J (v1.51) and MaxQuant search engine (v.1.5.2.8)

For manuscripts utilizing custom algorithms or software that are central to the research but not yet described in published literature, software must be made available to editors and reviewers. We strongly encourage code deposition in a community repository (e.g. GitHub). See the Nature Portfolio guidelines for submitting code & software for further information.

Data

Policy information about availability of data

All manuscripts must include a data availability statement. This statement should provide the following information, where applicable:

- Accession codes, unique identifiers, or web links for publicly available datasets
- A description of any restrictions on data availability
- For clinical datasets or third party data, please ensure that the statement adheres to our policy

Publicly available data used in this paper were obtained from Nephroseq (https://www.nephroseq.org/), a single-cell sequencing database (http://humphreyslab.com/SingleCell/) and UniProt FASTA database (https://www.uniprot.org/). For the data generated in this study, the RNA sequencing data has been submitted to National Center for Biotechnology Information (NCBI) Sequence Read Archive (SRA) database with the identifier PRJNA794204 (https://www.ncbi.nlm.nih.gov/bioproject/PRJNA794204/). Phosphoproteomic data of DNA-PKcs knockout and control kidney tissues has been submitted to iProX with the

identifier PXD030789 (http://proteomecentral.proteomexchange.org/cgi/GetDataset?ID=PXD030789). Mass spectrometry analysis of in vitro kinase assay has also been submitted to iProX with the identifier PXD036930 (http://proteomecentral.proteomexchange.org/cgi/GetDataset?ID=PXD036930). Metabolomics data of kidney tissues have been submitted to metabolights (www.ebi.ac.uk/metabolights/MTBLS5971). All the data of this study are available within the article, the Supplementary Information file, the Source data file, as described in the Reporting summary of this article. Source data are provided with this study.

Human research participants

Policy information about studies involving human research participants and Sex and Gender in Research.

Reporting on sex and gender	In this study, total 15 injured kidney samples obtained from patients with renal fibrosis (Both male and female patients were recruited, the clinical parameters of the patients are listed in Supplementary Table 1) and healthy kidney samples used for immunostaining studies were obtained from the Children's Hospital of Nanjing Medical University, China. 4 healthy control samples (include male and female) were nondiseased portions of tissue from renal cell carcinoma (RCC) patients who had undergone surgery to remove tumor tissues.
Population characteristics	clinical parameters of the patients are listed in Supplementary Table 1 of Supplementary Information
Recruitment	In this study, the kidney samples of patients with renal fibrosis were collected between 2019 and 2020 at Children's Hospital of Nanjing Medical University. The patients who were diagnosed with renal fibrosis at Children's Hospital of Nanjing Medical University between 2019 and 2020 were all recruited, all the patients (their parents/guardians) agreed to participation for free, so self-selection bias is low.
Ethics oversight	The protocol concerning the use of human kidney biopsy samples in this study was approved by the Committee on Research Ethics of Children's Hospital of Nanjing Medical University and informed consent was obtained from all human study participants (or their parents/guardians)

Note that full information on the approval of the study protocol must also be provided in the manuscript.

Field-specific reporting

Please select the one below that is the best fit for your research. If you are not sure, read the appropriate sections before making your selection.

☒ Life sciences ☐ Behavioural & social sciences ☐ Ecological, evolutionary & environmental sciences

For a reference copy of the document with all sections, see nature.com/documents/nr-reporting-summary-flat.pdf

Life sciences study design

All studies must disclose on these points even when the disclosure is negative.

Sample size	The number of samples for each assay was indicated in each figure legend. For in vitro cellular and biochemical assays, the sample sizes (at least three biological replicates) were chosen based on previous publications (PMID: 33181155, PMID: 25295536) with these assays yielding statistically significant difference between experimental positive and negative controls and on similar sample sizes. Results are representatives of at least three biological replicates. For in vivo assays, 5-6 mice were chosen for each condition, this sample size was determined by using power calculation for a t-test difference between two independent means based on a normally distributed population with equal variance.
Data exclusions	No data exclusions in this study.
Replication	A minimum of independent triplicates were carried out for each experiments as described in the legends for each figure.
Randomization	For in vivo, mice were randomly divided into experimental groups. For in vitro studies, samples were also randomly divided into different experimental groups.
Blinding	Because the same individual is involved in the animal and cell experiments, blinding was not used for these works. But the Investigators were blinded during the sample collection and data analysis （WB, PAS, IHC, MASSON and Sirius red staining etc.）

Reporting for specific materials, systems and methods

We require information from authors about some types of materials, experimental systems and methods used in many studies. Here, indicate whether each material, system or method listed is relevant to your study. If you are not sure if a list item applies to your research, read the appropriate section before selecting a response.

Materials & experimental systems

n/a	Involved in the study
	☒ Antibodies
	☒ Eukaryotic cell lines
☒	Palaeontology and archaeology
	☒ Animals and other organisms
☒	Clinical data
☒	Dual use research of concern

Methods

n/a	Involved in the study
☒	ChIP-seq
☒	Flow cytometry
☒	MRI-based neuroimaging

Antibodies

Antibodies used

WB antibodies:
Rabbit monoclonal anti-DNA-PKcs (Abcam, Cat:ab32566, clone: Y393, lot:GR3206273-15,dil 1/1000)
Rabbit polyclonal anti-DNA-PKcs (phospho S2056) (Abcam,Cat:ab18192,lot:GR3177522-4,dil 1/1000)
Rabbit polyclonal anti-Fibronectin(Abcam, Cat:ab2413,lot:GR3247081-1,dil 1/1000)
Rabbit monoclonal anti-Cas9 (S. pyogenes) (Cell Signaling Technology,Cat:19526, clone: E7M1H, lot:1,dil 1/1000)
Rabbit monoclonal anti-a-Smooth Muscle Actin (Cell Signaling Technology,Cat:19245,clone: D4K9N, lot:3,dil 1/1000)
Rabbit monoclonal anti-Raptor (Cell Signaling Technology,Cat:2280, clone: 24C12, lot:13,dil 1/1000)
Rabbit monoclonal anti-mTOR (phospho S2448) (Cell Signaling Technology,Cat:5536, clone: D9C2, lot:9,dil 1/1000)
Rabbit polyclonal anti-mTOR (Cell Signaling Technology,Cat:2972,lot:10,dil 1/1000)
Mouse monoclonal anti-TAF7 (TAFII P55) (Santa Cruz Biotechnology,Cat:sc101167, clone: SQ-8, lot:G1921,dil 1/1000)
Mouse monoclonal anti-Flag (Sigma-Aldrich,Cat:F1804, clone: M2, lot:1003310512,dil 1/1000)
Mouse monoclonal anti-GAPDH (Proteintech,Cat:60004-1-Ig, clone: 1E6D9, lot:10017731,dil 1/1000)
Mouse monoclonal anti-beta-ACTIN (Proteintech,Cat:66009-1-Ig, clone: 2D4H5, lot:10021787,dil 1/1000)
Rabbit polyclonal anti-ACOX1 (Proteintech,Cat:10957-1-AP,lot:00079141,dil 1/1000)
Rabbit polyclonal anti-CPT1A (Proteintech,Cat:66039-1-Ig,lot:10003772,dil 1/1000)
Rabbit polyclonal anti-LDHA(Proteintech,Cat:19987-1-AP,lot:00102198,dil 1/1000)
Mouse monoclonal anti-HK2 (Proteintech,Cat:66974-1-Ig, clone: 2A11C3, lot:10008341,dil 1/1000)
Rabbit polyclonal anti-Lamin B1 (Proteintech,Cat:12987-1-AP,lot:00092016,dil 1/1000)
Rabbit polyclonal anti-FIS1(Proteintech,Cat:10956-1-AP,lot:00113446,dil 1/1000)
Mouse monoclonal anti-Alpha Tubulin (Proteintech,Cat: 66031-1-Ig, clone: 1E4C11, lot:10004185,dil 1/1000)

Goat anti-rabbit IgG-HRP (Beyotime,Cat:A0208,dil 1/1000)
Goat anti-mouse IgG-HRP (Beyotime,Cat:A0216,dil 1/1000)

IHC/IF antibodies:
Rabbit monoclonal anti-DNA-PKcs (human)(Abcam, Cat:ab133516, clone: EPR392, lot:GR3259087-11,dil 1/100)
Rabbit polyclonal anti-DNA-PKcs (phospho S2056) (Abcam,Cat:ab18192, lot:GR3177522-4,dil 1/100)
Rabbit monoclonal anti-DNA-PKcs (Abcam, Cat:ab32566, clone: Y393, lot:GR3206273-15,dil 1/100)
Mouse monoclonal anti-a-Smooth Muscle Actin(IF Formulated) (Cell Signaling Technology,Cat:48938, clone: 1A4, lot:2,dil 1/200)
Rabbit monoclonal anti- COL1A1 (Cell Signaling Technology,Cat:72026, clone: E8F4L, lot:1,dil 1/100)
Rabbit monoclonal anti-F4/80 (Cell Signaling Technology,Cat:70076, clone: D2S9R, lot:8,dil 1/150)
Rabbit monoclonal anti-Histone H2A.X (Ser139) (Cell Signaling Technology,Cat:9718, clone: 20E3, lot:21,dil 1/200)
Mouse monoclonal anti-TAF7 (TAFII P55) (Santa Cruz Biotechnology,Cat:sc101167, clone: SQ-8, lot:G1921,dil 1/50)
Lotus tetragonolobus lectin (LTL) (Vector lab, Cat: FL1321, lot: ZH0805, dil 1/200)
Donkey anti-Rabbit IgG (H+L) Highly Cross-Adsorbed Secondary Antibody, Alexa Fluor 488 (Thermo Fisher Scientific,Cat:21206,lot:2289872,dil 1/500)
Donkey anti-Rabbit IgG (H+L) Highly Cross-Adsorbed Secondary Antibody, Alexa Fluor™ Plus 555 (Thermo Fisher Scientific,Cat:A32794,dil 1/500)

IP/ChIP antibodies
Rabbit polyclonal anti-DNA-PKcs (Abcam, Cat:ab70250, lot:GR3359077-11,dil 1/100)
Rabbit monoclonal anti-FLAG (ChIP)(Cell Signaling Technology, cat: 14793; clone: D6W5B, lot 3)
Rabbit mAb IgG Isotype Control (Cell Signaling Technology, cat.n.3900, clone: DA1E, lot 4),
Mouse mAb IgG Isotype Control (Cell Signaling Technology, cat.n.5415; clone: G3A1, lot 4).

Validation

DNA-PKcs(Abcam, Cat:ab32566)
https://www.abcam.cn/dna-pkcs-antibody-y393-ab32566.html
DNA-PKcs (phospho S2056)(Abcam,Cat:ab18192)
https://www.abcam.cn/dna-pkcs-phospho-s2056-antibody-ab18192.html
human DNA-PKcs (Abcam, Cat:ab133516)
https://www.abcam.cn/dna-pkcs-antibody-epr392-ab133516.html
Fibronectin(Abcam, Cat:ab2413)
https://www.abcam.cn/fibronectin-antibody-ab2413.html
Cas9 (S. pyogenes) (Cell Signaling Technology,Cat:19526)
https://www.cellsignal.cn/products/primary-antibodies/cas9-s-pyogenes-e7m1h-xp-rabbit-mab/19526?site-search-type=Products&N=4294956287&Ntt=cas9+%28s.+pyogenes%29+%28e7m1h%29+xp&fromPage=plp
α-Smooth Muscle Actin (Cell Signaling Technology,Cat:19245)
https://www.cellsignal.cn/products/primary-antibodies/a-smooth-muscle-actin-d4k9n-xp-rabbit-mab/19245?site-search-type=Products&N=4294956287&Ntt=19245&fromPage=plp&_requestid=2667654

α-Smooth Muscle Actin (1A4) Mouse mAb (IF Formulated) (Cell Signaling Technology,Cat:48938)
https://www.cellsignal.cn/products/primary-antibodies/a-smooth-muscle-actin-1a4-mouse-mab-if-formulated/48938?site-search-type=Products&N=4294956287&Ntt=48938&fromPage=plp&_requestid=2667972
COL1A1 (Cell Signaling Technology,Cat:72026,lot:1,dil 1/100)
https://www.cellsignal.cn/products/primary-antibodies/col1a1-e8f4l-xp-rabbit-mab/72026?site-search-type=Products&N=4294956287&Ntt=72026&fromPage=plp&_requestid=2668227
Raptor (Cell Signaling Technology,Cat:2280)
https://www.cellsignal.cn/products/primary-antibodies/raptor-24c12-rabbit-mab/2280?site-search-type=Products&N=4294956287&Ntt=2280&fromPage=plp&_requestid=2668548
mTOR (phospho S2448) (Cell Signaling Technology,Cat:5536)
https://www.cellsignal.cn/products/primary-antibodies/phospho-mtor-ser2448-d9c2-xp-rabbit-mab/5536?site-search-type=Products&N=4294956287&Ntt=5536&fromPage=plp&_requestid=2668690
mTOR (Cell Signaling Technology,Cat:2972)
https://www.cellsignal.cn/products/primary-antibodies/mtor-antibody/2972?site-search-type=Products&N=4294956287&Ntt=2972&fromPage=plp&_requestid=2668916
F4/80 (Cell Signaling Technology,Cat:70076)
https://www.cellsignal.cn/products/primary-antibodies/f4-80-d2s9r-xp-rabbit-mab/70076?site-search-type=Products&N=4294956287&Ntt=70076&fromPage=plp&_requestid=2669141
Histone H2A.X (Ser139) (Cell Signaling Technology,Cat:9718)
https://www.cellsignal.cn/products/primary-antibodies/phospho-histone-h2a-x-ser139-20e3-rabbit-mab/9718?site-search-type=Products&N=4294956287&Ntt=9718&fromPage=plp&_requestid=2669856
TAF7 (TAFII P55) (Santa Cruz Biotechnology,Cat:sc101167)
https://www.scbt.com/p/taf-ii-p55-antibody-sq-8?requestFrom=search
Flag (Sigma-Aldrich,Cat:F1804)
https://www.sigmaaldrich.cn/CN/zh/search/f1804?focus=products&page=1&perpage=30&sort=relevance&term=f1804&type=product
GAPDH (Proteintech,Cat:60004-1-Ig)
https://www.ptgcn.com/products/GAPDH-Antibody-60004-1-Ig.htm
beta-ACTIN (Proteintech,Cat:66009-1-Ig)
https://www.ptgcn.com/products/Pan-Actin-Antibody-66009-1-Ig.htm
ACOX1 (Proteintech,Cat:10957-1-AP)
https://www.ptgcn.com/products/AOX-Antibody-10957-1-AP.htm
CPT1A (Proteintech,Cat:66039-1-Ig)
https://www.ptgcn.com/products/CPT1A-Antibody-66039-1-Ig.htm
LDHA (Proteintech,Cat:19987-1-AP)
https://www.ptgcn.com/products/LDHA-Specific-Antibody-19987-1-AP.htm
HK2 (Proteintech,Cat:66974-1-Ig)
https://www.ptgcn.com/products/HK2-Antibody-66974-1-Ig.htm
Alpha Tubulin (Proteintech,Cat:66031-1-Ig)
https://www.ptgcn.com/products/tubulin-Alpha-Antibody-66031-1-Ig.htm
Lamin B1(Proteintech,Cat:12987-1-AP)
https://www.ptgcn.com/products/LMNB1-Antibody-12987-1-AP.htm
FIS1(Proteintech,Cat:10956-1-AP)
https://www.ptgcn.com/products/FIS1-Antibody-10956-1-AP.htm
DNA PKcs (Abcam, Cat:ab70250)
https://www.abcam.cn/dna-pkcs-antibody-ab70250.html
FLAG (Cell Signaling Technology, cat: 14793)
https://www.cellsignal.cn/products/primary-antibodies/dykddddk-tag-d6w5b-rabbit-mab-binds-to-same-epitope-as-sigma-s-anti-flag-m2-antibody/14793?site-search-type=Products&N=4294956287&Ntt=14793&fromPage=plp&_requestid=2675123

Eukaryotic cell lines

Policy information about cell lines and Sex and Gender in Research

Cell line source(s)	Human renal tubular epithelial cells (HK-2), mouse renal proximal tubular cells (mPTCs), normal rat kidney interstitial fibroblasts (NRK-49F) and Human Embryonic Kidney 293 (HEK293T) were obtained from American Type Culture Collection (ATCC)
Authentication	Cell lines were authenticated by short tandem repeat (STR) analysis.
Mycoplasma contamination	Cell lines were not tested for mycoplasma contamination but no indication of contamination was observed
Commonly misidentified lines (See ICLAC register)	No commonly misidentified cell lines were used.

Animals and other research organisms

Policy information about studies involving animals; ARRIVE guidelines recommended for reporting animal research, and Sex and Gender in Research

Laboratory animals	DNA-PKcs knockout (DNA-PKcs-/-) mice on a BALB/c background were generated and purchased from GemPharmatech (Nanjing, China). DNA-PKcs-/- mice and wild-type (WT) littermates were bred in the Laboratory Animal Center of Nanjing Medical University

	(Nanjing, China).To construct proximal renal tubular epithelial cells-specific Cas9 transgenic mice, Rosa26-floxed STOP-Cas9 knockin mice (purchased from GemPharmatech, C57BL/6 background) and Kap-icre mice were purchased from Jackson. WT mice (C57BL/6J) were purchased from GemPharmatech. All the mice used in this study for UUO or UIR were male mice of approximately 6-8 week. Mice were housed in pathogen free and ventilated cages, and allowed free access to irradiated food and auto claved water ad libitum in a 12h light/dark cycle, with room temperature at 21±2°C and humidity between 45 and 65%.
Wild animals	No wild animals were used in this study.
Reporting on sex	In general, the reason for using male mice in animal experiments is to avoid interference with the female estrous cycle. Additionally, since female hormones can affect the fibrosis progression resulting in variations. Therefore, only male mice were used in our animal studies.
Field-collected samples	No Field-collected samples were used.
Ethics oversight	Animal procedures were approved by the Institutional Animal Care and Use Committee of Nanjing Medical University.

Note that full information on the approval of the study protocol must also be provided in the manuscript.
